# Curved optical solitons subject to transverse acceleration in reorientational soft matter

**DOI:** 10.1038/s41598-017-12242-5

**Published:** 2017-09-28

**Authors:** Urszula A. Laudyn, Michał Kwaśny, Filip A. Sala, Mirosław A. Karpierz, Noel F. Smyth, Gaetano Assanto

**Affiliations:** 1 Warsaw University of Technology, Faculty of Physics, Warsaw, 00-662 Poland; 2 University of Edinburgh, School of Mathematics, Edinburgh, EH9 3FD Scotland UK; 3 University of Rome “Roma Tre”, NooEL— Nonlinear Optics and OptoElectronics Lab, Rome, 00146 Italy; 4 Tampere University of Technology, Photonics Lab, Tampere, FI 33101 Finland

## Abstract

We demonstrate that optical spatial solitons with non-rectilinear trajectories can be made to propagate in a uniaxial dielectric with a transversely modulated orientation of the optic axis. Exploiting the reorientational nonlinearity of nematic liquid crystals and imposing a linear variation of the background alignment of the molecular director, we observe solitons whose trajectories have either a monotonic or a non-monotonic curvature in the observation plane of propagation, depending on either the synergistic or counteracting roles of wavefront distortion and birefringent walk-off, respectively. The observed effect is well modelled in the weakly nonlinear regime using momentum conservation of the self-collimated beams in the presence of the spatial nonlocality of the medium response. Since reorientational solitons can act as passive waveguides for other weak optical signals, these results introduce a wealth of possibilities for all-optical signal routing and light-induced photonic interconnects.

## Introduction

Nematic liquid crystals (NLCs) are among the prime examples of soft matter; due to the rod-like structure of the organic molecules, they exhibit optical and electric anisotropy^1^. Within a specific range of temperatures, in which they exist in the nematic phase, the long axes of the molecules are nearly parallel to one another and their average alignment is described by a dimensionless unit vector **n**, termed the molecular director. Most NLCs are optically uniaxial with positive birefringence (with the extraordinary refractive index larger than the ordinary one) with optic axis along **n**. One of the most important features of NLCs is that their orientation, resulting from long-range intermolecular interactions in the fluid state, can be easily modified by electric and/or magnetic fields^1,2^. Light-induced changes in the molecular alignment give rise to a large reorientational optical nonlinearity, characterized by a diffusive-like nonlocal response which extends beyond the size of the electromagnetic (beam) disturbance. The reorientation of light-induced molecular dipoles towards the electric field vector **E** is driven by a torque which tends to minimize the point-wise system energy. Such reorientation increases the refractive index *n*_*e*_ of extraordinary waves towards the largest plane-wave eigenvalue ${n}_{\parallel } > {n}_{\perp }$, leading to self-focusing^2,3^. When the input wavepacket is intense enough so that the latter Kerr-like response balances linear diffraction, the beam can form a spatial solitary wave or soliton^4^. Optical spatial solitons are diffractionless light beams able to confine themselves and guide other optical signals in self-focusing media^5^. In reorientational dielectrics, specifically nematic liquid crystals, they are termed nematicons and can be generated at low power excitations (even sub-mW) over extended propagation distances (several mm), only limited by scattering losses^6–9^. Nematicons are stable and robust self-confined extraordinary-polarized wavepackets; they exhibit long-range mutual attraction and survive interactions with interfaces, dielectric perturbations and external stimuli^10–14^. Moreover, being able to guide copolarized signals at shorter or longer wavelengths^15–17^, nematicons are excellent candidates for real time light controlled guided wave photonics^18,19^. Since curved waveguides are desirable elements in photonics, hereby we investigate the propagation of nematicons which, at variance with their straight propagation in uniform NLCs, bend in NLC structures subject to a transverse modulation of the director distribution. In such samples, at variance with the use of geometric phases through polarization evolution^20,21^, their transverse velocity is not a constant owing to the combined action of wavefront curvature and birefringent walk-off. The experimental results of this paper, obtained in samples prepared by electron-beam lithography and in excellent agreement with a simple model based on momentum conservation, demonstrate that various nematicon curvatures can be achieved by introducing a linear modulation of the director orientation across one of the transverse coordinates and selecting the launch location of the input beam. Both monotonic and non-monotonic trajectory curvatures can result from appropriate combinations of background orientation and modulation of the optic axis distribution.

## Nematicons

Nematicons are optical spatial solitary waves in positive uniaxial nematic liquid crystals, birefringent materials characterized by different refractive indices for electric fields parallel and perpendicular to the optic axis **n**. The equilibrium position of the director in the absence of external stimuli (including a light beam) is then determined by the anchoring conditions at the boundaries of the cell containing the nematic through intermolecular links^1^. When a finite light beam with electric field **E** propagates as an extraordinary wave in the principal plane of homogeneously oriented NLCs, i.e., the plane defined by the optic axis and the wavevector **k**, upon reorientation from the initial angle ${\theta }_{0}=\angle {\bf{k}}{\bf{n}}$ in the absence of the optical beam to the new value *θ*, the refractive index undergoes an increase and acquires a pointwise value

1 $${n}_{e}=\frac{{n}_{\parallel }{n}_{\perp }}{\sqrt{{n}_{\perp }^{2}\,{\sin }^{2}\,\theta +{n}_{\parallel }^{2}\,{\cos }^{2}\,\theta }}.$$

The latter yields self-focusing and, eventually, a refractive potential able to confine the beam into a solitary wave. The inherent birefringence is also linked to the angular departure–the walkoff *δ*–of the e-wave energy flux, that is the Poynting vector **S**, from the wave vector **k** (see Methods).

Hence, nematicons are walking solitons, i.e., self-trapped extraordinarily polarized wavepackets which uniformly propagate in the principal (**n**, **k**) plane with transverse velocity *δ* with respect to **k** ^22^.

**Figure 1:**
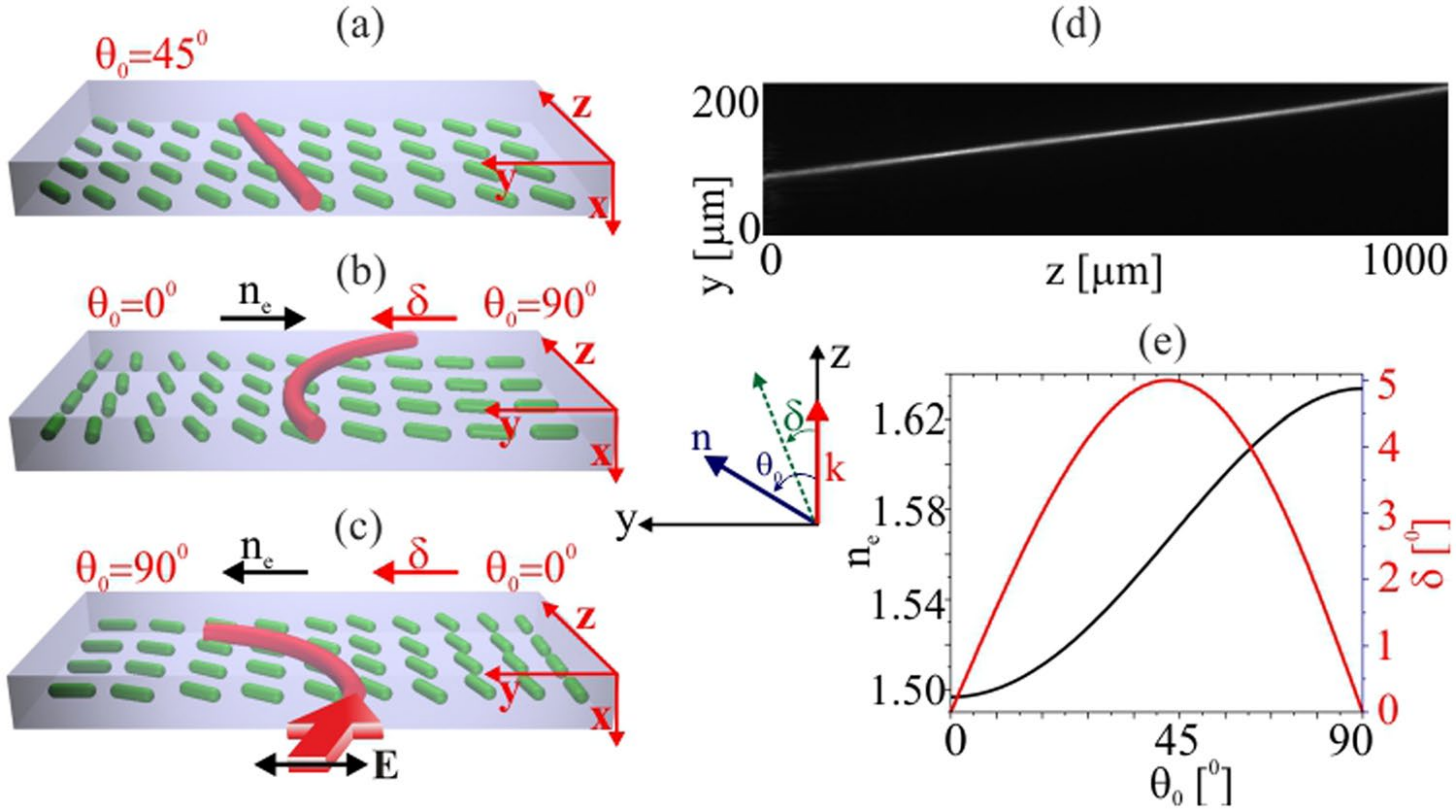
(**a**) Sketch of a planar cell with uniform initial orientation *θ*_0_ = 45°. The inset defines the main quantities and their mutual orientation. (**b**) Sketch of a cell with transverse modulation in director orientation (0°–90° across *y*) when the refractive index change and walk-off act together; (**c**) director orientation (90°–0° across *y*) when refraction and walk-off compete. The Gaussian input beam is linearly polarized along *y*; red and black arrows indicate the trajectory bending in the principal plane owing to walk-off and refraction, respectively. (**d**) Acquired evolution in (*y*, *z*) of an extraordinary-wave nematicon of power *P* = 2 *mW* in a cell with uniform orientation *θ*_0_ = 45°, as in (**a**); (**e**) Calculated walk-off (red line) and refractive index (black line) in 6CHBT versus initial orientation *θ*_0_ at *λ* = 1.064 *μm*.

Several approaches have been undertaken to modify the walk-off of nematicons by altering the director orientation *θ* of the liquid crystal molecules, including voltage induced reorientation through the electro-optic response of NLC^23–28^, the anchoring conditions at the boundaries^29^, self-induced reorientation in the highly nonlinear regime^30–33^ and external magnetic fields^34,35^. Nematicon trajectories have also been modified by creating refractive perturbations due to external beams^36–39^, interfaces^11,38,39^ and other nematicons^10,12,14,40–42^. Additional routing strategies rely on tunnelling^43,44^, total internal reflection^11,39,45^ and the use of chiral NLCs^46–49^. Hereby, we investigate a nematicon with a non-rectilinear trajectory propagating in non-uniformly oriented NLCs in the simplest case of a linear modulation of the background angle *θ*_0_ across the transverse coordinate *y* in the principal plane (**n**, **k**) (see Fig. 1). The geometry we consider therefore encompasses longitudinal invariance, a Gaussian monochromatic input beam with electric field polarized along *y* and a wavevector **k** along *z*, an in-plane director distribution in (*y*, *z*) and various director angles *θ*_0_ at the edges of the cell, such that refractive index distribution and walk-off *δ* combine to bend the soliton path in either the same or opposite directions.

## Results and Discussion

In order to implement a dielectric configuration as described above, we used a 30 *μm* thick layer of nematic liquid crystals with planar anchoring at the inner interfaces of a glass cell. A number of planar NLC samples were prepared according to the geometries sketched in Fig. 1(a–c). We consider the propagation of a linearly polarized bell-shaped and monochromatic light beam. The extraordinary wave beam initially propagates along *z*, with its electric field **E** oscillating in the transverse *y* direction (see inset in Fig. 1). Figure 1(a) shows a planar sample with a uniform initial orientation, with cell interfaces rubbed so that the molecular director forms an angle *θ*_0_ = 45° with *z* in the (*y*, *z*) plane. Such an orientation avoids the Freéderiksz transition and maximizes the nonlinear response of the medium^50,51^. Figure 1(b,c) presents cases with an additional y-dependent rotation of the angle *θ* i.e. a linearly varying orientation of the optic axis across the transverse coordinate *y* in the principal plane. This linear variation influences the refractive index for extraordinary waves, as well as the walk-off. Within this simple configuration of a transversely non-uniform director arrangement, two regimes were investigated: (i) trajectories when the increase of the walk-off and refractive index counteract (Fig. 1(b)); (ii) trajectories when the walk-off and refraction act in the same direction (Fig. 1(c)). A *y*-polarized Gaussian beam of wavelength *λ* = 1.064 *μm* was focused at the entrance of the planar sample and launched in the midplane (half thickness), coupling to extraordinary waves with electric field oscillating in the plane (*y*, *z*). At powers near or above 3 mW, the input beam could excite a nematicon. The trajectories were monitored along *z* in the (*y*, *z*) plane for various launch locations, corresponding to various initial orientations *θ*_0_. In NLCs with a uniform director distribution and beam wavevector ${\bf{k}}\parallel z$, the nematicon trajectory is determined only by the walk-off, which leads to a rectilinear path in the principal plane, as shown in Fig. 1(d) for a typical experiment with the NLC mixture 6CHBT (see the Methods section). Figure 1(e) shows the calculated extraordinary-wave refractive index and walk-off versus the initial (at rest) director alignment *θ*_0_ for the investigated material. For orientations close to *θ*_0_ = 45° the walk-off reaches its maximum of 5° at room temperature and wavelength *λ* = 1.064 *μm*. Noteworthy, even though the light beam produces an extra point-wise rotation of *θ* by means of the nonlinear response (enabling it to self-focus and form a nematicon^6–9^), in the weak nonlinear regime of interest here the beam walk-off remains determined only by the background orientation value *θ*_0_^22,23,31^.

**Figure 2:**
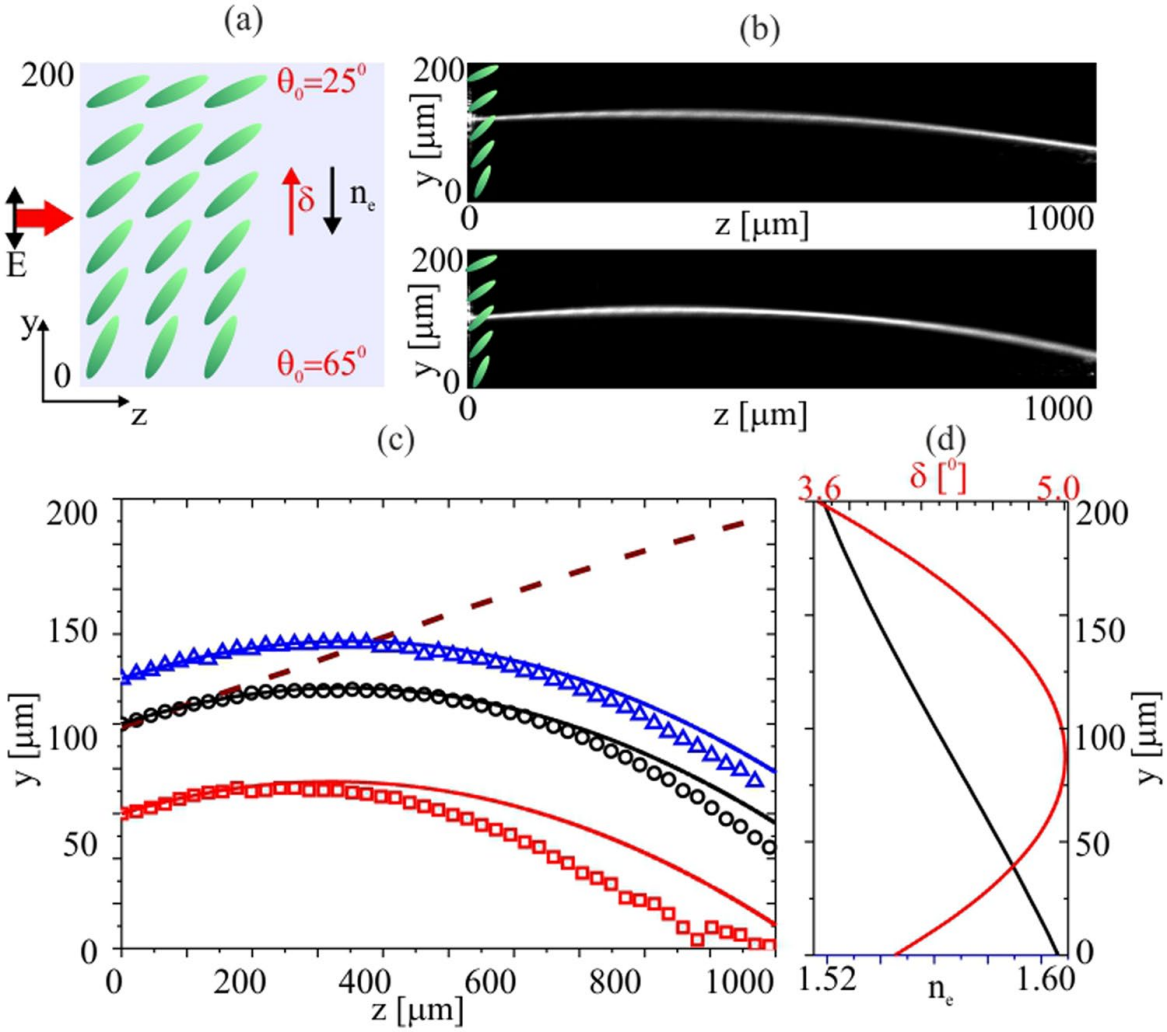
(**a**) Sketch of (*y*, *z*) orientation *θ*_0_ ranging from 65° to 25° across *y*. (**b**) Images of nematicon evolution in the (*y*, *z*) plane of observation for a *P* = 3 *mW* beam launched in *z* = 0 at *y* = 120 *μm* (*θ*_0_ = 45°) and at *y* = 100 *μm* (*θ*_0_ = 41°), upper and lower photographs, respectively. (**c**) Experimentally acquired (symbols) and theoretical (solid lines) nematicon trajectories for various input positions across *y*; the dashed line indicates the trajectory in uniform NLCs with *θ*_0_ = 45°. (**d**) Calculated walk-off (red line) and refractive index across *y* for *λ* = 1.064 *μm* in 6CHBT at room temperature.

We first studied the effects of walk-off and refraction on the nematicon path for the case of a director alignment varying at the linear rate 40°/200 *μm* across the cell width. Figure 2 shows the experimental results obtained in the cell with the orientation ranging from 65° (*y* = 0) to 25° (*y* = 200 *μm*). The resulting nematicon path has an initial walk-off angle ranging from 3.6° up to 5° and back to 4° with respect to *z* as the refractive changes from 1.52 up to 1.60 (Fig. 2(d)). The increase in refractive index is monotonic across *y* and causes beam refraction towards *y* = 0, whereas the initial walk-off tends to move the Poynting vector towards *y* = 200 *μm* (dashed line in Fig. 2(c)), with the walk-off and wavefront distortion counteracting along the cell (Fig. 2(a)). The beams initial direction is then affected by the transverse modulation of the director orientation, with the transverse slope in the refractive index distribution counteracting the walk-off, eventually leading to a reversed transverse velocity and a nematicon bending with a trajectory exhibiting a maximum in the (*y*, *z*) plane (Fig. 2(b,c)). The non-uniform orientation has a significant impact on beam propagation after a distance of *z* = 200 *μm*. Figure 2(c) shows the experimentally recorded nematicon paths (symbols) and the theoretical beam trajectories given by momentum conservation for various input positions across *y*, according to the modulated walk-off and refractive index distributions (Fig. 2(d)). For the simulations, a simple model based on momentum conservation^52^ was implemented and its results used for the nematicon trajectories (see the Methods section).

**Figure 3:**
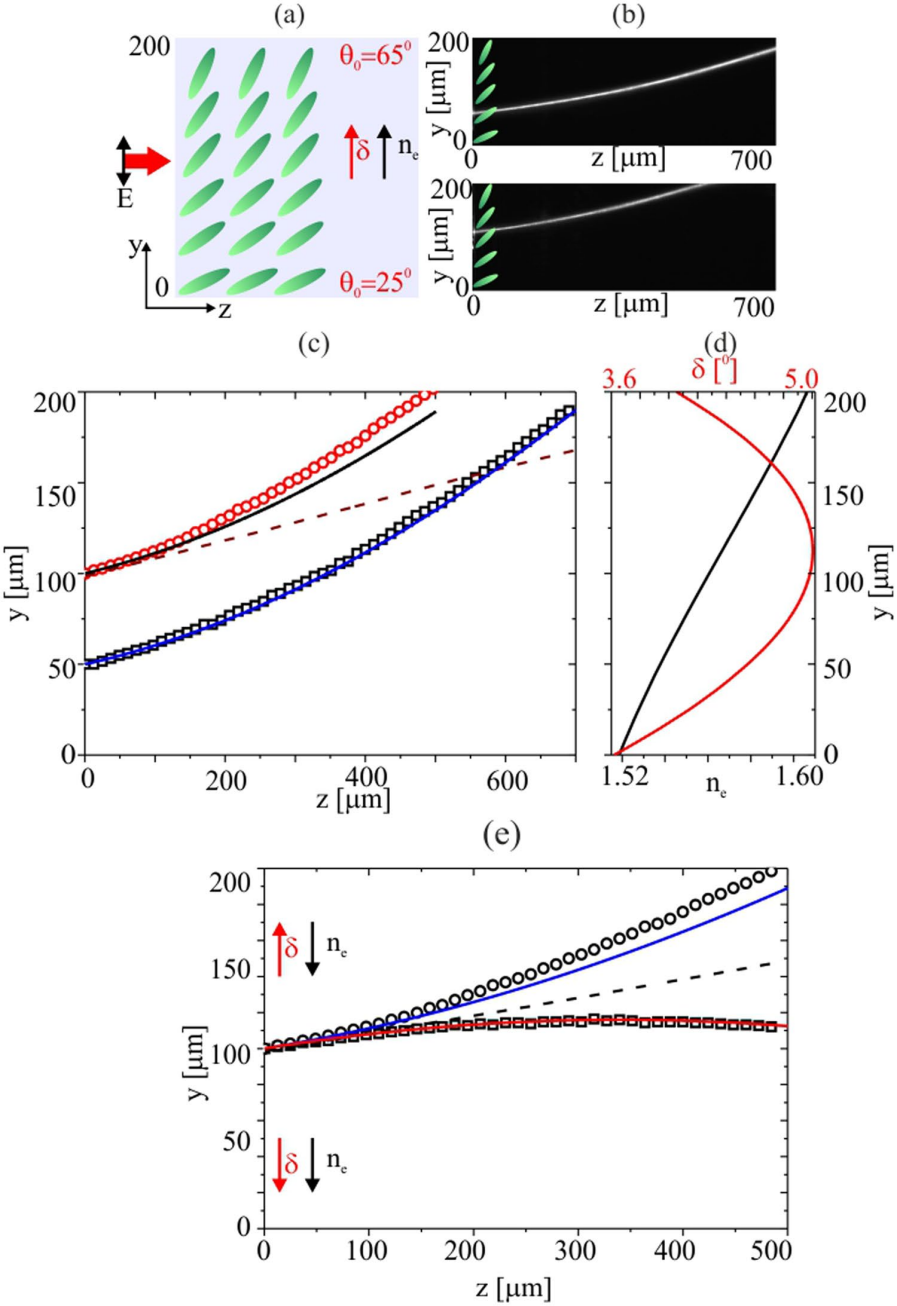
(**a**) Sketch of (*y*, *z*) orientation *θ*_0_ ranging from 25° to 65° across *y* when refraction and walk-off cooperate. (**b**) Nematicon evolution in (*y*, *z*) for a *P* = 3 *mW* beam launched at *z* = 0 at *y* = 50 *μm* (*θ* = 35°, upper image) and at *y* = 100 *μm* (*θ* = 45°, lower image). (**c**) Experimentally acquired (symbols) and theoretical (solid lines) nematicon trajectories for different input positions across *y*; the brown dashes indicate the path for the uniform case *θ*_0_ = 45°. (**d**) Calculated walk-off (red line) and refractive index across *y* for *λ* = 1.064 *μm* in 6CHBT at room temperature. (**e**) Comparison of nematicon trajectories in (*y*, *z*) when refractive index change and walk-off compete (squares, red line) and cooperate (circles, blue line) for beams launched in *z* = 0 at *y* = 100 *μm*; the symbols are the measured paths, the solid lines are the theoretical simulations; the dashed line is the rectilinear trajectory in a uniform sample with *θ*_0_ = 45°.

Figure 3 shows the results obtained in the case of the refraction and the walk-off both bending the beam towards *y* = 200 *μm*, with the orientation angle linearly varying from 25° to 65° (Fig. 3(a)). Figure 3(b) shows the acquired trajectories for two different transverse locations of the input beam. Figure 3(c) displays the experimentally recorded (symbols) and theoretical trajectories for two input positions across *y*; the corresponding walk-off and transverse refractive index distributions are displayed in Fig. 3(d). In this case the role of the non-uniform orientation becomes important after the first 150 *μm* of propagation, i.e., earlier than in Fig. 2 owing to the synergistic contributions of the walk-off and phase-front distortion. In Fig. 3(e) we compare nematicon trajectories–both measured and theoretical–for cases of walk-off and refraction acting together (blue line, squares) or in opposition (red line, circles), respectively, for an input beam launched at *y* = 100 *μm* (*θ*_0_ = 45°). The dashed line gives the trajectory for a uniform orientation *θ*_0_ = 45°.

**Figure 4:**
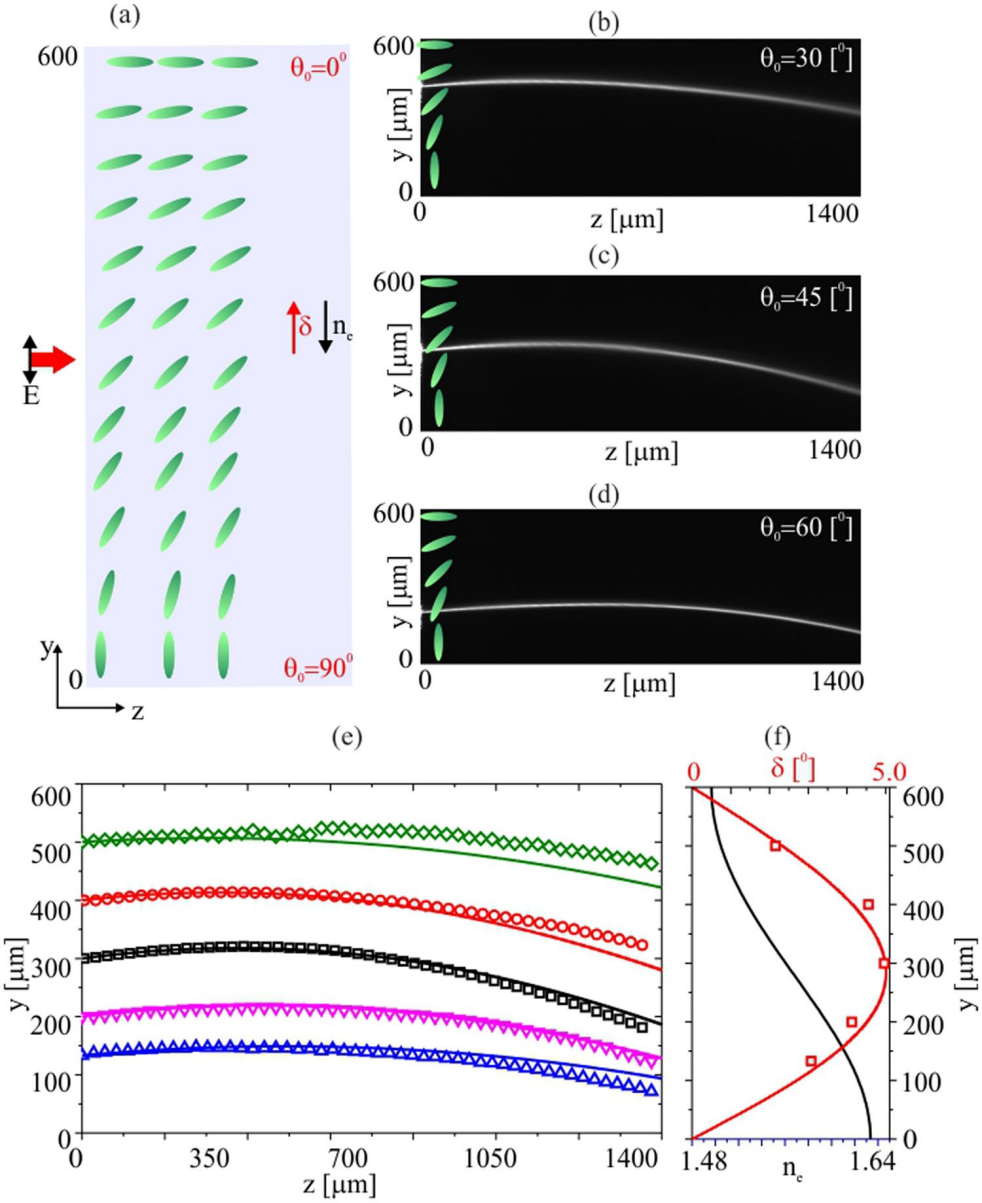
(**a**) Geometry with orientation angle *θ*_0_ ranging from 90° to 0° across the 600 *μm* width. (**b**–**d**) Images of the acquired beam evolution in (*y*, *z*) for *P* = 3 *mW* and various input position across *y*. (**e**) Comparison of experimental (symbols) and theoretical (solid lines) data for trajectories for different launch locations. (**f**) Computed walk-off (red line) and refractive index (black line) across *y*; the red squares correspond to the measured initial walk-off (between *z* = 0 and *z* = 100 *μm*).

**Figure 5:**
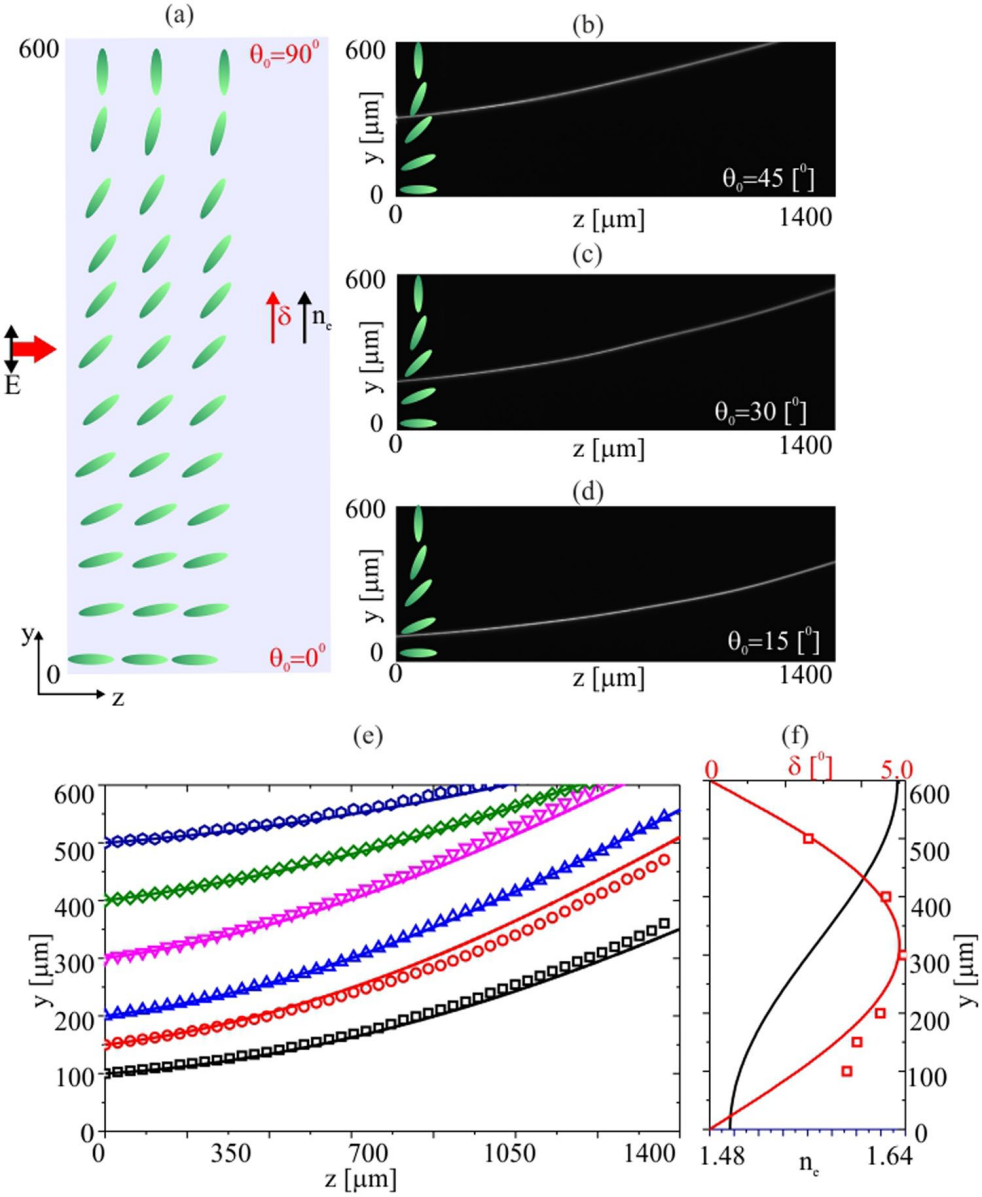
(**a**) Arrangement of the cell with orientation ranging from 0 to 90° across *y*. (**b**–**d**) Acquired evolution of 3 *mW* nematicons for three input beam positions and orientations. (**e**) Comparison between experimental (symbols) and theoretical (solid lines) trajectories for various launch locations. (**f**) Calculated walk-off (red line) and refractive index (black line) across *y*; the red squares indicate the measured initial walk-off.

To further enhance the nematicon bending, we prepared planar cells with the molecular director anchored at a linearly modulated *θ* ranging from 90° to 0° between *y* = 0 to *y* = 600 *μm*, respectively. The resulting initial propagation direction (Poynting vector direction) ranged from *δ* = 0° to *δ* = 5°, depending on the launch position across *y*. Again, we investigated two cases, when walk-off and refractive index changes counteract (Fig. 4) and cooperate (Fig. 5), respectively.

Figure 4 presents the cell geometry (Fig. 4(a)) and acquired beam evolution along *z* for three input locations across *y* (Fig. 4(b–d)). As expected, the nematicon trajectory bends differently when launching the wavepacket at different initial orientations *θ*_0_. Figure 4(e) displays the experimentally measured trajectories for various input positions and the corresponding results from the theoretical modelling. Over the first 100 *μm* of propagation, the nematicon walks off at an angle given by the initial director orientation (Fig. 4(f)). After this, the modulated alignment along *y* plays a significant role and the nematicon trajectory curves towards the region of higher refractive index, counteracting the walk-off. The bending is enhanced at the launch position *y* = 300 *μm* where the molecules are initially oriented at 45° as the phase-front distortion is strongest there and the walk-off is close to its maximum.

In the opposite case, with both the refractive index and walk-off acting in the same direction, the nematicon trajectories bend even more, as expected. Figure 5(a) displays the cell arrangement and nematicon evolution in (*y*, *z*) for three launch locations (Fig. 5(b–d)). Similarly to the previous case (Fig. 4), the strongest deviation is for an input at *y* = 300 *μm*, i.e., where the initial orientation is *θ*_0_ = 45°. Figure 4(e) shows the experimental and calculated trajectories. As for the case of Fig. 4, from *z* = 0 to *z* = 100 *μm* the nematicon propagates at an angle corresponding to the initial walk-off (squares, Fig. 5(f)).

**Figure 6:**
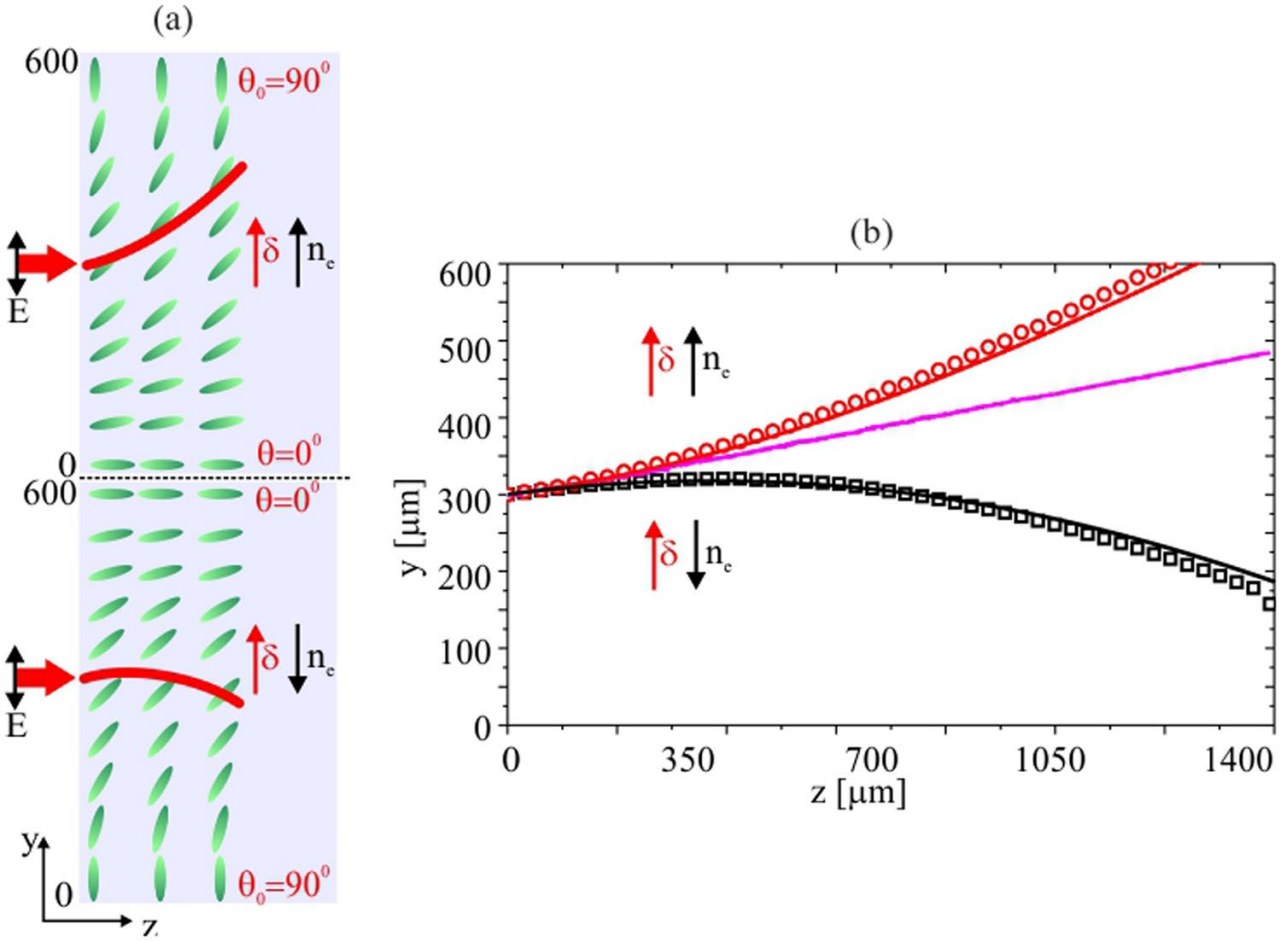
(**a**) Orientation ranging from 90° to 0° and back to 90° across *y* in a double width cell geometry. (**b**) Measured (symbols) and theoretical nematicon trajectories when launched where *θ*_0_ = 45° for which refraction and walk-off cooperate (red symbols and line, upper cell) or counteract (black symbols and line, lower cell). The magenta solid line corresponds to the uniform case with *θ*_0_ = 45° everywhere.

Finally, Fig. 6 summarizes nematicon trajectories for the two cases presented above for a beam launched at *y* = 300 *μm*, i.e., with *θ*_0_ = 45°. When refraction acts in conjunction with walk-off, the beam trajectory deviation from a straight line is enhanced. In the opposite case, the nematicon trajactory reverses its transverse velocity and eventually bends towards the region with higher refractive index. Even in the simplest limit of linear modulation of the orientation angle, the nematicon trajectory and its associated waveguide can have both a monotonic curvature or an inversion of the transverse velocity, depending on the director distribution.

## Conclusions

We have investigated the propagation of reorientational optical spatial solitary waves (nematicons) in nematic liquid crystals in the presence of a transversely varying alignment of the optic axis. We specifically addressed the case of linear modulation of the anchoring applied at the boundaries of planar cells, comparing regimes with cooperating and counteracting phase front distortion (refraction) and birefringent walk-off. We demonstrated that the nematicon trajectories bend according to physical intuition. In addition, a simple model based on momentum conservation for the nematicon was found to give a trajectory solution in excellent agreement with these experimental results. This shows the power of theoretical modelling based on solitary wave perturbation theory. These results pave the way for the engineering of light-induced optical waveguides, well beyond the standard limitation of straight trajectories, as imposed by homogeneous media.

## Methods

### Experimental measurements and sample preparation

For the experiments we used linearly polarized Gaussian (*TEM*_00_) beams from a Nd:YAG laser operating at *λ* = 1.064 *μ*m and linearly polarized with electric field along *y*, focused by a microscope objective (20×) at the input of the cell (*z* = 0) to a waist *w*_0_ ≈ 3 *μ*m in the midplane between upper and lower glass/NLC interfaces. The beam propagation in the principal plane (*y*, *z*) was monitored with a high-resolution CCD camera, imaging the light which was Rayleigh scattered out of the observation (principal) plane. The sample position and alignment of the laser were adjusted by precise 3-axis micro-translation stages.

Planar glass cells were prepared with 1.1 mm thick BK7 glass slides covered with Indium-Tin-Oxide; the propagation length (along *z*) was 1 to 1.4 mm, the thickness— defined by the slide separation across *x*— was 30 *μ*m. Upper and lower glass slides were cleaned in an ultrasonic bath and then spin coated with Polymethyl-Methyl-Acrylate at 1000 rpm before baking them on a hot plate at 160 °C for 60 s. Finally, they were treated by electron beam lithography (10 *kV* voltage with 30 *μ*m aperture and 110 *μCcm* ^−2^ irradiation) in order to define the director anchoring at the prescribed angle distribution *θ*_0_^53^. The latter process led to the decomposition of the polymer bonds, so that after irradiation the substrates were immersed in a 1:3 solution of Methyl Isobutyl Ketone with Isopropyl Alcohol for 30 s, then rinsed and blow-dried with Nitrogen. As a result, line and space grooves were obtained with width and separation of 500 *nm*. The UV-curable photopolymer NOA-61 mixed with 30 *μ*m glass spacers was placed over one of the substrates before assembling the cell and exposing it to UV light. The assembled cells were then filled up with 6CHBT^54^ by capillarity, avoiding the formation of air bubbles/gaps near the boundaries and checking the director alignment under a polarization microscope.

The highly birefringent nematic liquid crystal we employed was 6CHBT (synthesized by Prof. R. Dabrowski at the Military University of Technology, Poland^54^), with refractive indices *n*_⊥_ = 1.4967 and ${n}_{\parallel }=1.6335$ at room temperature and *λ* = 1.064 *μ*m, Frank elastic constants *K*_11_ = 8.96 *pN*, *K*_22_ = 3.61*pN* and *K*_33_ = 9.71 *pN* for splay, twist and bend deformations, respectively, and a transition temperature *T*_*c*_ = 43 °C.

### Modelling and momentum conservation

The experimental and theoretical results were compared in the main text and figures, showing excellent agreement. Here we outline the derivation of the analytical solution, based on momentum conservation for the beam. A more detailed account of the theoretical model and its validity is given in ref.^52^.

We consider the propagation of a linearly polarised, extraordinary (e-) wave, bell-shaped beam of wavelength *λ* and power *P* through bulk uniaxial nematic liquid crystals with non-uniform orientation of the optic axis. The polarisation of the input electric field *E* of the beam is parallel to the *y* direction and its propagation is along the *z* axis, with *x* completing the coordinate triad. The beam is inputted with wavevector along *z* at *y* = *y*_*in*_, where the director orientation is *θ*_*in*_. The orientation angle has an additional linear modulation *θ*_*b*_ across the cell width, with slope ${\tilde{\theta }}_{b}^{^{\prime} }$. Through the reorientational nonlinear response, the injected e-wave beam rotates the molecular director by an additional angle *ϕ*, so that the director locally makes the total angle *θ* = *θ*_*in*_ + *θ*_*b*_ + *ϕ* = *θ*_0_ + *ϕ* to *z* in the presence of an e-wave optical wavepacket. The extraordinary refractive index is given by

2 $${n}_{e}^{2}(\theta )=\frac{{n}_{\perp }^{2}{n}_{\parallel }^{2}}{{n}_{\parallel }^{2}\,{\cos }^{2}\,\theta +{n}_{\perp }^{2}\,{\sin }^{2}\,\theta },$$

where ${n}_{\parallel }$ and *n*_⊥_ are the eigenvalues for plane waves polarised parallel and perpendicular to the molecular director (optic axis), respectively. The optical anisotropy is then ${\rm{\Delta }}\varepsilon ={n}_{\parallel }^{2}-{n}_{\perp }^{2}$. The equations for beam propagation will be written in the single constant approximation, in which the elastic constants for bend, twist and splay deformations are all equal, *K* = *K*_11_ = *K*_22_ = *K*_33_. For simplicity, the equations in the non-uniform nematic liquid crystals will be set in non-dimensional form in the coordinate system (*X*, *Y*, *Z*) and the electric field *u*, where

3 $$x=WX,\quad y=WY,\quad z=BZ,\quad {E}_{y}=Au.$$

The relations between the physical and the non-dimensional variables are

4 $$W=\frac{\lambda }{\pi \sqrt{{\rm{\Delta }}\epsilon \,\sin \,2{\theta }_{in}}},\quad B=\frac{2{n}_{e}\lambda }{\pi {\rm{\Delta }}\epsilon \,\sin \,2{\theta }_{in}},\quad {A}^{2}=\frac{2P}{\pi {\rm{\Gamma }}{W}^{2}},\quad {\rm{\Gamma }}=\frac{1}{2}{\epsilon }_{0}c{n}_{e}.$$

The equations governing the propagation of the beam in the non-uniform reorientational dielectric are then

5 $$i\frac{\partial u}{\partial Z}+i\gamma {\rm{\Delta }}({\theta }_{0})\frac{\partial u}{\partial Y}+\frac{1}{2}{\nabla }^{2}u+2({\theta }_{0}+\varphi )\,u=\mathrm{0,}$$

6 $$\nu {\nabla }^{2}\varphi =-2{|u|}^{2}.$$

The coefficient Δ is related to the birefringent walk-off *δ* of the extraordinary-wave beam, with tan *δ* = Δ, and is given by

7 $${\rm{\Delta }}(\theta )=\frac{{\rm{\Delta }}\epsilon \,\sin \,2\theta }{{\rm{\Delta }}\epsilon +2{n}_{\perp }^{2}+{\rm{\Delta }}\epsilon \,\cos \,2\theta }.$$

The walkoff coefficient *γ* in the electric field equation (5), arising from the non-dimensionalisation, is

8 $$\gamma =\frac{2{n}_{e}}{\sqrt{{\rm{\Delta }}\epsilon \,\sin \,2{\theta }_{in}}}.$$

The non-dimensional elastic constant *ν* in the director equation (6) is given by

9 $$\nu =\frac{8K}{{\varepsilon }_{0}{\rm{\Delta }}\epsilon {A}^{2}{W}^{2}\,\sin \,2{\theta }_{in}}.$$

Finally, in these dimensionless variables the imposed linear variation in the background director orientation is

10 $${\theta }_{b}(Y)={\theta }_{in}+{\theta }_{b}^{^{\prime} }(Y-{\xi }_{in}),$$

as (in the non-dimensional system) the beam is launched at *Y* = *ξ*_*in*_ at *Z* = 0. The non-dimensional slope of the linear director variation is ${\theta }_{b}^{^{\prime} }$.

The trajectory of a nematicon in non-uniform nematic liquid crystals will be determined using “momentum conservation” for the model equations (5) and (6). The term momentum is used here as a mechanical analogy, as in solitary wave perturbation theory^55^. The simplest approach to determine the model resulting from this momentum conservation approximation is from the Lagrangian formulation of the equations (5) and (6). The Lagrangian for this system is

11 $$L=i({u}^{\ast }{u}_{Z}-u{u}_{Z}^{\ast })+i\gamma {\rm{\Delta }}({\theta }_{0})\,({u}^{\ast }{u}_{Y}-u{u}_{Y}^{\ast })-{|\nabla u|}^{2}+4\,({\theta }_{0}+\varphi )\,{|u|}^{2}-\nu {|\nabla \varphi |}^{2},$$

where the $\ast$ superscript denotes the complex conjugate. Solitary wave perturbation theory is usually based on a (slowly varying) version of the exact solitary wave solution for a given nonlinear dispersive wave equation^55^. However, the equations (5) and (6) have no known exact solitary wave solutions in a uniform medium with *θ*_*b*_ = 0, only specific solutions for fixed parameter values^56^. Fortunately, the highly nonlocal response of liquid crystals can be invoked^9^, so that this solitary wave solution is not needed. Let us take the solitary wave solution of the nematic liquid crystal equations to be

12 $$u=ag(\rho ){e}^{i\sigma +iV(Y-\xi )}\quad {\rm{and}}\quad \varphi =\alpha {g}^{2}(\mu ),$$

where

13 $$\rho =\frac{\sqrt{{X}^{2}+{(Y-\xi )}^{2}}}{w},\quad \mu =\frac{\sqrt{{X}^{2}+{(Y-\xi )}^{2}}}{\beta }.$$

The exact functional form *g* of the solitary wave solution is not specified, but is chosen radially symmetric. Solitary wave perturbation theory then proceeds by assuming that the solitary wave parameters *a*, *w*, *σ*, *V*, *ξ*, *α* and *β* are functions of *Z* ^55^. Now, it was found in previous work^38,57–59^ that the trajectory of a nematicon in the nonlocal limit is independent of its profile variations, that is its amplitude and width evolution. As we are only interested in the trajectory of the nematicon in this work, we can assume that the amplitudes *a* and *α* and widths *w* and *β* are fixed at their initial values and independent of *Z*.

Substituting the profile forms (12) into the Lagrangian (11) and averaging by integrating in *X* and *Y* from −∞ to ∞^60^ gives the averaged Lagrangian^60,61^

14 $$\begin{array}{rcl} {\mathcal L}  & = & -2{S}_{2}(\sigma ^{\prime} -V\xi ^{\prime} )\,{a}^{2}{w}^{2}-{S}_{22}{a}^{2}-{S}_{2}\,({V}^{2}+2V{F}_{1}-4F)\,{a}^{2}{w}^{2}\\  &  & +\frac{2{A}^{2}{B}^{2}\alpha {\beta }^{2}{a}^{2}{w}^{2}}{{A}^{2}{\beta }^{2}+{B}^{2}{w}^{2}}-4\nu {S}_{42}{\alpha }^{2}-2q{S}_{4}{\alpha }^{2}{\beta }^{2},\end{array}$$

where primes denote differentiation with respect to *Z*. Here *F* and *F*_1_, which determine the beam trajectory, are expressed by

15 $$F(\xi )=\frac{{\int }_{-\infty }^{\infty }\,{\int }_{-\infty }^{\infty }\,{\theta }_{0}{g}^{2}\,dXdY}{{\int }_{-\infty }^{\infty }\,{\int }_{-\infty }^{\infty }\,{g}^{2}\,dXdY},$$

16 $${F}_{1}(\xi )=\frac{{\int }_{-\infty }^{\infty }\,{\int }_{-\infty }^{\infty }\,\gamma {\rm{\Delta }}\,({\theta }_{0})\,{g}^{2}\,dXdY}{{\int }_{-\infty }^{\infty }\,{\int }_{-\infty }^{\infty }\,{g}^{2}\,dXdY}.$$

The integrals *S*_2_, *S*_4_ and *S*_22_ and *S*_42_ in this averaged Lagrangian are

17 $$\begin{array}{ll}{S}_{2}={\int }_{0}^{\infty }\,\zeta {g}^{2}(\zeta )\,d\zeta , & {S}_{22}={\int }_{0}^{\infty }\,\zeta {g}^{^{\prime} 2}(\zeta )\,d\zeta ,\\ {S}_{4}={\int }_{0}^{\infty }\,\zeta {g}^{4}(\zeta )\,d\zeta , & {S}_{42}=\frac{1}{4}{\int }_{0}^{\infty }\,\zeta {[\frac{d}{d\zeta }{g}^{2}(\zeta )]}^{2}\,d\zeta .\end{array}$$

Taking variations of the averaged Lagrangian (14) with respect to *ξ* and *V* yields the modulation equations

18 $$\frac{dV}{dZ}=2\frac{dF}{d\xi }-V\frac{d{F}_{1}}{d\xi },$$

19 $$\frac{d\xi }{dZ}=V+{F}_{1},$$

which determine the beam trajectory. These equations are mechanical momentum equations for a point particle of mass 1 and “velocity” *V* evolving with “time” *Z*.

These momentum equations cannot be solved as the integrals (15) and (16) cannot be evaluated without knowledge of the nematicon profile *g*. To obtain a solution, we now approximate these integrals in the nonlocal limit, as the nematicon width 3 *μm* is much less than the length scale of the refractive index variation, which is 300 *μm* as ${\theta }_{b}^{^{\prime} }\sim 0.003\,rad/\mu m$. Then

20 $$F(\xi )\sim {\theta }_{0}(\xi ),\quad {F}_{1}(\xi )\sim \gamma {\rm{\Delta }}({\theta }_{0}(\xi \mathrm{))}.$$

A further approximation is based on the imposed linear variation *θ*_*b*_ in the background angle being much less than *θ*_*in*_^52^. The expression for *F*_1_ in (20) can then be expanded in a Taylor series about *θ*_*in*_,

21 $${F}_{1}(\xi )=\gamma {\rm{\Delta }}({\theta }_{in})+\gamma {\rm{\Delta }}^{\prime} ({\theta }_{in})\,{\theta }_{b}(\xi )+\cdots $$

With these approximations, the trajectory (momentum) equations (18) and (19) become

22 $$\frac{dV}{dZ}=(2-V\gamma {\rm{\Delta }}^{\prime} ({\theta }_{in}))\,{\theta }_{b}^{^{\prime} }(\xi ),$$

23 $$\frac{d\xi }{dZ}=V+\gamma {\rm{\Delta }}({\theta }_{in})+\gamma {\rm{\Delta }}^{\prime} ({\theta }_{in})\,{\theta }_{b}(\xi \mathrm{)}.$$

Thanks to these approximations, the final momentum equations have the exact solution for the linear variation *θ*_*b*_

24 $$\begin{array}{rcl}\xi  & = & [{\xi }_{in}+\frac{1+{\gamma }^{2}{\rm{\Delta }}^{\prime} ({\theta }_{in})\,{\rm{\Delta }}({\theta }_{in})}{{\gamma }^{2}{{\rm{\Delta }}}^{^{\prime} 2}({\theta }_{in})\,{\theta }_{b}^{^{\prime} }}]\,{e}^{\gamma {\rm{\Delta }}^{\prime} ({\theta }_{in}){\theta }_{b}^{^{\prime} }Z}-\frac{2+{\gamma }^{2}{\rm{\Delta }}^{\prime} ({\theta }_{in})\,{\rm{\Delta }}({\theta }_{in})}{{\gamma }^{2}{{\rm{\Delta }}}^{^{\prime} 2}({\theta }_{in})\,{\theta }_{b}^{^{\prime} }}\\  &  & \,\,+\frac{1}{{\gamma }^{2}{{\rm{\Delta }}}^{^{\prime} 2}({\theta }_{in})\,{\theta }_{b}^{^{\prime} }}{e}^{-\gamma {\rm{\Delta }}^{\prime} ({\theta }_{in}){\theta }_{b}^{^{\prime} }Z}\end{array}$$

as ${\theta }_{b}^{^{\prime} }$ is a constant. This expression for the nematicon trajectory was compared with the experimental results.
